# Supplementary figures and images for: Complete chloroplast genome of a montane plant Spathoglottis aurea Lindl.: Comparative analyses and phylogenetic relationships among members of tribe collabieae
Source: PLoS One. 2024 Sep 16;19(9):e0291888. doi: 10.1371/journal.pone.0291888 (PMC11404822; doi:10.1371/journal.pone.0291888)

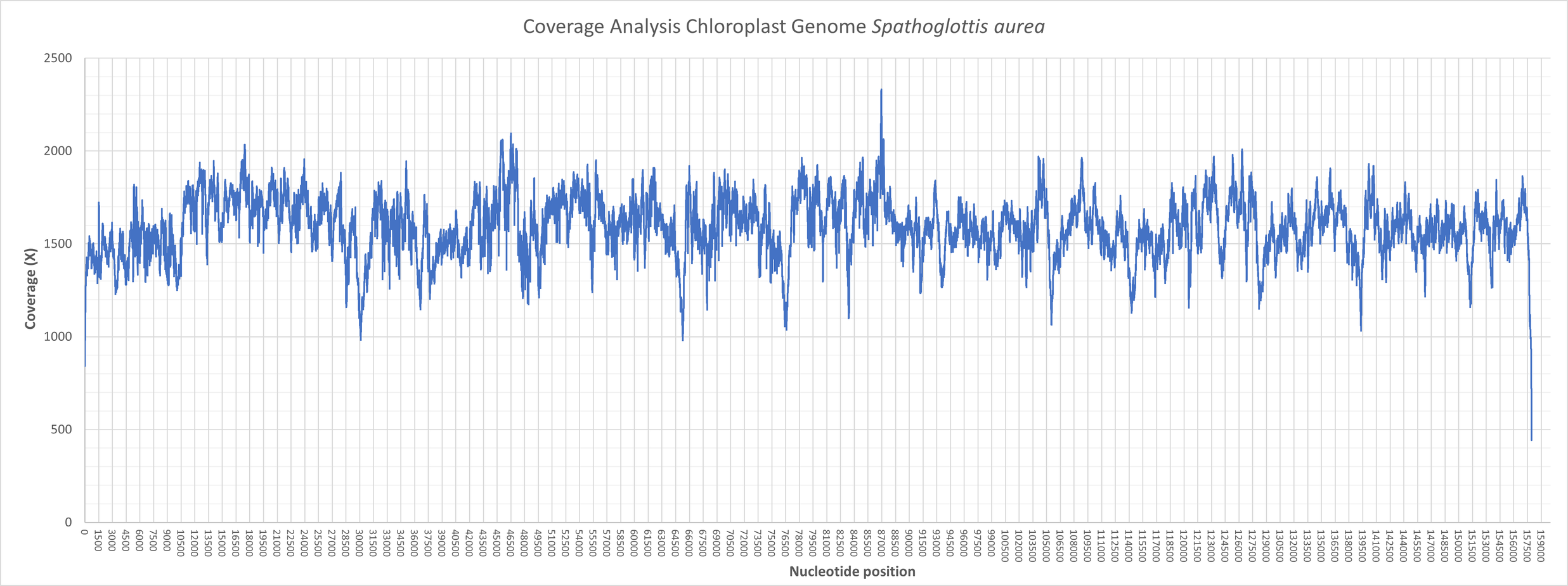

Supplement: S1 Fig — Coverage depth of nucleotide position of S. aurea chloroplast genome. (TIF) [file pone.0291888.s001.tif]
