# Supplementary material for: Complete chloroplast genome of a montane plant Spathoglottis aurea Lindl.: Comparative analyses and phylogenetic relationships among members of tribe collabieae
Source: PLoS One. 2024 Sep 16;19(9):e0291888. doi: 10.1371/journal.pone.0291888 (PMC11404822; doi:10.1371/journal.pone.0291888)
Supplement: S1 Table — Codon usage table S. aurea chloroplast genome. (DOCX) [file pone.0291888.s002.docx]

**S1 Table.** Codon usage table Spathoglottis aurea chloroplast genome

| **Amino Acid** | **Codon** | **Frequency** | **RSCU** | **Fraction** | **Amino Acid** | **Codon** | **Frequency** | **RSCU** | **Fraction** |
| --- | --- | --- | --- | --- | --- | --- | --- | --- | --- |
| Ala  (A) | GCG | 112 | 0.4 | 0.1 | Pro  (P) | CCG | 90 | 0.43 | 0.12 |
|  | GCA | 336 | 1.2 | 0.3 |  | CCA | 226 | 1.08 | 0.3 |
|  | GCT | 513 | 1.83 | 0.46 |  | CCT | 324 | 1.55 | 0.39 |
|  | GCC | 160 | 0.57 | 0.14 |  | CCC | 194 | 0.93 | 0.19 |
| Cys  (C) | TGT | 182 | 1.56 | 0.78 | Gln  (Q) | CAG | 175 | 0.47 | 0.26 |
|  | TGC | 51 | 0.44 | 0.22 |  | CAA | 574 | 1.53 | 0.74 |
| Asp  (D) | GAT | 675 | 1.62 | 0.81 | Arg  (R) | AGG | 131 | 0.6 | 0.1 |
|  | GAC | 157 | 0.38 | 0.19 |  | AGA | 420 | 1.93 | 0.33 |
| Glu  (E) | GAG | 285 | 0.49 | 0.25 |  | CGG | 91 | 0.42 | 0.07 |
|  |  |  |  |  |  | CGA | 287 | 1.32 | 0.22 |
|  | GAA | 871 | 1.51 | 0.75 |  | CGT | 300 | 1.38 | 0.22 |
|  |  |  |  |  |  | CGC | 76 | 0.35 | 0.06 |
| Phe  (F) | TTT | 738 | 1.28 | 0.64 | Ser  (S) | AGT | 320 | 1.22 | 0.2 |
|  | TTC | 414 | 0.72 | 0.36 |  |  |  |  |  |
|  |  |  |  |  |  | AGC | 93 | 0.36 | 0.06 |
| Gly  (G) | GGG | 244 | 0.69 | 0.17 |  | TCG | 132 | 0.5 | 0.08 |
|  | GGA | 551 | 1.56 | 0.39 |  | TCA | 309 | 1.18 | 0.20 |
|  | GGT | 461 | 1.31 | 0.33 |  | TCT | 465 | 1.78 | 0.3 |
|  | GGC | 153 | 0.43 | 0.11 |  | TCC | 251 | 0.96 | 0.16 |
| His  (H) | CAT | 394 | 1.55 | 0.78 | Thr  (T) | ACG | 115 | 0.44 | 0.11 |
|  | CAC | 144 | 0.45 | 0.22 |  | ACA | 315 | 1.2 | 0.3 |
|  |  |  |  |  |  | ACT | 429 | 1.63 | 0.41 |
| Ile  (I) | ATA | 523 | 0.91 | 0.30 |  | ACC | 192 | 0.73 | 0.18 |
|  | ATT | 842 | 1.46 | 0.49 |  |  |  |  |  |
|  | ATC | 362 | 0.63 | 0.21 | Val  (V) | GTG | 180 | 0.62 | 0.16 |
|  |  |  |  |  |  | GTA | 418 | 1.44 | 0.36 |
| Lys  (K) | AAG | 287 | 0.5 | 0.25 |  | GTT | 408 | 1.41 | 0.35 |
|  | AAA | 857 | 1.5 | 0.75 |  | GTC | 154 | 0.53 | 0.13 |
| Leu  (L) | TTG | 433 | 1.23 | 0.20 | Trp  (W) | TGG | 364 | 1 | 1 |
|  | TTA | 658 | 1.86 | 0.31 |  |  |  |  |  |
|  | CTG | 149 | 0.42 | 0.07 | Tyr  (Y) | TAT | 575 | 1.59 | 0.80 |
|  | CTA | 307 | 0.87 | 0.14 |  | TAC | 148 | 0.41 | 0.20 |
|  | CTT | 434 | 1.23 | 0.20 |  |  |  |  |  |
|  | CTC | 137 | 0.39 | 0.06 | End  (stop codon) | TGA | 19 | 0.8 | 0.27 |
|  |  |  |  |  |  | TAG | 21 | 0.89 | 0.30 |
| Met  (start codon) | ATG | 488 | 1 | 1 |  | TAA | 31 | 1.31 | 0.44 |
| Asn  (N) | AAT | 764 | 1.57 | 0.78 |  |  |  |  |  |
|  | AAC | 211 | 0.43 | 0.22 |  |  |  |  |  |

*Average codon = 20,690
